# Epidemiology, clinical profile and treatment patterns of venous thromboembolism in cancer patients in Taiwan: a population-based study

**DOI:** 10.1186/s12885-015-1200-6

**Published:** 2015-04-17

**Authors:** Tan-Wei Chew, Churn-Shiouh Gau, Yu-Wen Wen, Li-Jiuan Shen, C Daniel Mullins, Fei-Yuan Hsiao

**Affiliations:** 1 Graduate Institute of Clinical Pharmacy, College of Medicine, National Taiwan University, R220, 33, Linsen S. Road, Taipei, 10050 Taiwan; 2 School of Pharmacy, College of Medicine, National Taiwan University, Taipei, Taiwan; 3 Center for Drug Evaluation, Taipei, Taiwan; 4 Clinical Informatics and Medical Statistics Research Center, Chang Gung University, Tao-Yuan, Taiwan; 5 Department of Pharmacy, National Taiwan University Hospital, Taipei, Taiwan; 6 University of Maryland School of Pharmacy, Baltimore, MD USA

**Keywords:** Venous thromboembolism, Cancer, Epidemiology, Population-based study

## Abstract

**Background:**

Venous thromboembolism (VTE) is a clinically significant complication that is well documented among Caucasian cancer patients. However, evidence regarding VTE incidence and treatment among Asian cancer patients is very limited. The objective of this study is to investigate the incidence, risk factors and management of VTE among Taiwanese cancer patients.

**Methods:**

Using Taiwan’s National Health Insurance Research Database, we identified 43,855 newly diagnosed cancer patients between 2001 and 2008. Two alternative algorithms for identifying VTE event were explored to better quantify a range of incidence rates of VTE in our cancer patients. Multivariable logistic regression models were used to explore VTE risk factors.

**Results:**

The incidence rates of VTE were 9.9 (algorithm 1) and 3.4 (algorithm 2) per 1,000 person-years, respectively. The incidence rates were higher in certain cancers, particularly liver, pancreas, and lung. Significant risk factors for VTE were site of cancer, prior history of VTE, chemotherapy and major surgeries. Long-term anticoagulant therapy was initiated in 64.1% patients with VTE and 72.2% of them received warfarin alone. Approximately two-thirds of patients with VTE received ≤ 3 months of anticoagulant therapy.

**Conclusion:**

Incidence of cancer-related VTE is lower among Taiwanese compared to Caucasian populations. Nevertheless, risk factors for cancer-related VTE found in our study were consistent with current literature.

## Background

Venous thromboembolism (VTE) is a significant complication among cancer patients. The incidence rates of VTE among Caucasian cancer patients were reported to be 4-20%. Cancer patients have 4- to 7-folds higher risk for VTE than the general population [1,2]. In addition, VTE-associated complications such as bleeding events, post-thrombotic syndrome and recurrence of VTE complicate the clinical management of cancer and worsen patients’ quality of life [2]. Existing studies have further linked VTE to a higher risk of 1-year death post cancer diagnoses [3,4]. Several professional organizations, including American Society of Clinical Oncology (ASCO) and National Comprehensive Cancer Network (NCCN), have therefore issued guidelines regarding treatment and prophylaxis of VTE among cancer patients [5-9]. However, these guidelines were based on data mainly from Caucasian populations and their applications in different racial/ethnic populations are left unanswered.

In particular, available information on the epidemiology of VTE among Asian cancer patients is very limited. Although observational studies have tried to fill this knowledge gap, most existing studies were limited by small sample sizes and specific cancer sites [10-15]. Different methodological approaches may have contributed to dissimilar estimates of the incidence of VTE in the Asian cancer patients as well. Furthermore, treatment patterns for VTE among the Asian population may not follow clinical guidelines. A large scale, epidemiological study can help us to understand the incidence and treatment of VTE among Asian cancer patients and optimize clinical practice. Using a nationally-representative dataset, we conducted a population-based cohort study to investigate the epidemiology, risk factors, and clinical profile of VTE among Taiwanese cancer patients. In addition, we examined VTE treatment patterns in this population.

## Methods

### Data source

The data source of this population-based cohort study was the Taiwan’s National Health Insurance research database (NHIRD). The NHIRD is a nationwide database comprising demographic data, clinical data, medical resource utilization data (outpatient and inpatient visits), costs of services, and treatment patterns of more than 99% of the entire population (23 million) in Taiwan. All traceable personal identifiers are removed from the database to protect patient privacy. The database has been described in detail elsewhere [16]. The NHIRD has been maintained since 1997 and has been used to conduct many population-level studies [16,17]. Three subsets of the NHIRD, the Longitudinal Health Insurance Health Insurance Database 2000 (LHID 2000), 2005 (LHID 2005) and 2010 (LHID 2010), which contains claims data of one-million beneficiaries randomly selected from the Registry of Beneficiaries of the NHIRD in 2000, 2005, and 2010, respectively. The LHID 2000, LHID 2005, and LHID 2010 thus include approximately 15% of the total population in Taiwan. The databases used in this study included all inpatient and outpatient medical claims of the LHID 2000, LHID 2005 and LHID 2010 from January, 1999 to December, 2009.

### Ethical statement

Because the identification numbers for all of the subjects in the NHRID were encrypted to protect the privacy of the individuals, this study was exempt from a full review by the Institutional Review Board of the National Taiwan University Hospital and informed consent was waived.

### Study population

Newly diagnosed cancer patients defined by those who have been first-ever hospitalized with a primary diagnosis of malignant disease (International Classification of Diseases, Ninth Revision, Clinical Modification (ICD-9-CM) code (ICD-9-CM codes: 140–208)) between January 1, 2001 and December 31, 2008 were identified. A two-year wash-out period was applied to ensure their incident diagnoses of cancer. The date when a patient was first hospitalized with a primary diagnosis of malignant disease was defined as the index date. Cancer subtypes analyzed in this study included head and neck (ICD-9-CM codes: 140–149, 160–161), esophageal (150), stomach (151), colorectum (153–154), liver (155), pancreas (157), other abdominal (152, 156, 158–159), lung (162–163), sarcoma (170–171), skin (172–173), breast (174–175), endometrium and cervix (179–182), ovary (183), prostate (185), testis (186), bladder (188), renal (189), brain (191–192), thyroid (193), non-Hodgkin’s lymphoma (200, 202), Hodgkin’s lymphoma (201), multiple myeloma (203), and leukemia (204–208). Patients were excluded if their genders were unknown. Those who had more than one primary diagnosis of malignant diseases at index date were also excluded, for their cancer sites cannot be categorized.

### Identification of VTE

Two algorithms of VTE event were adopted in our study to better quantify a range of incidence rates of VTE in our cancer patients. VTE algorithm 1 was defined as a hospital admission with diagnostic codes of VTE (ICD-9-CM codes 415.1x, 451.xx, 452, and 453.xx). VTE algorithm 2 was based on both the hospital admission with diagnostic codes of VTE and managements of VTE (prescription of intravenous or subcutaneous (IV/SC) anticoagulants (unfractionated heparin (UFH) or low-molecular-weight heparin (LMWH)) or reimbursement codes of surgical thromboectomy) during the hospital stay.

### Comorbid diseases and potential risk factors for VTE

Comorbid diseases, including hypertension, heart failure, ischemic heart disease, atrial fibrillation, renal insufficiency, liver disease, chronic lung disease, diabetes mellitus, stroke, rheumatologic diseases, varicose veins of lower extremities, degenerative and paralytic neurologic disease, peripheral vascular disease, anemia, arterial embolism and obesity, were retrieved from both the outpatient and inpatient medical claims for 1 year before or during the index date using relevant ICD-9 CM codes. A history of VTE was defined as being hospitalized with VTE diagnosis within 2 years before the index date.

Potential risk factors of VTE, including pregnancy, major surgery, hospitalization, cancer treatments (chemotherapy (including biologic therapy), radiation therapy, hormone therapy, and combination therapy), major extremity trauma, major spine trauma, blood transfusion, and infectious disease, were retrieved from inpatient or outpatient medical claims from 3 months before the VTE event to the end of follow-up date.

### Treatment pattern of VTE

Among patients who had VTE events, we examined both initial and long-term treatment of VTE. Initial treatment patterns for patients who had VTE events were examined. Patients whose VTE events met VTE algorithm 2 were followed to see their long-term anticoagulant treatment pattern of VTE. Duration of long-term anticoagulant treatment was calculated from the discharge date of first hospitalization of VTE until the recurrence of VTE or end of follow-up date and was categorized into ≤ 3 months, 3–6 months, 6–12 months, and longer than 12 months.

### Statistical analysis

Crude incidence rates of VTE for the entire cancer patients and subgroups of patients categorized by sites of cancer were calculated as the number of cases per 1,000 person-years. For VTE cases, the follow-up time started from index date to the date of VTE event. For patients without VTE event, the follow-up time started from index date to the end of follow-up. Comparisons between cancer patients with and without VTE were performed using Student’s *t*-test for continuous variables and chi-square or Fisher’s exact test for discrete variables.

Multivariable logistic regression models using stepwise selection were carried out to identify risk factors for VTE for the cohort defined using algorithm 2. To assess the association between cancer site and risk of VTE, we regrouped cancer sites as those with higher risk of VTE (GI tracts (stomach, colorectum, pancreas, liver, and esophagus), brain, lung, endometrium and cervix, ovary, and kidney) [1], hematological malignant diseases (non-Hodgkin’s lymphoma, Hodgkin’s lymphoma, multiple myeloma, and leukemia) and other sites of cancer. Statistical significance was set at p < 0.05 and all tests were two-tailed. SAS software (Version 9.2; SAS Institute Inc., Cary, NC, USA) and Microsoft Office Excel 2010 were used in this study for the claims data conversion and analysis.

## Results

### Baseline characteristics of study cohort and incidence rate of VTE

We identified 43,855 newly diagnosed cancer patients between January 1, 2001 and December 31, 2008. The mean age (± SD) of the study cohort was 59.5 years (±15.9 years). Slightly more than half of them (52.7%) were men and nearly forty percent (41.9%) of patients were aged 65 years and older. Colorectal cancer (14.7%) was the most frequently diagnosed cancer in our study cohort, followed by breast cancer (13.8%), liver cancer (12.0%), head and neck cancer (10.0%), and lung cancer (9.5%).

Among 43,855 newly diagnosed cancer patients, hospital admissions for VTE (algorithm 1) were identified in 1,388 patients (3.2%) during or after index date. As shown in Table 1, the overall incidence rate of VTE (algorithm 1) was 9.88 per 1,000 person-years. The incidence rates of VTE were higher in men than women (13.56 vs. 6.61 per 1,000 person-years). The incidence rates of VTE were higher in certain cancers, particularly cancer of liver (68.23 per 1,000 person-years), pancreas (27.83 per 1,000 person-years), lung (17.22 per 1,000 person-years), multiple myeloma (10.56 per 1,000 person-years), and non-Hodgkin’s lymphoma (9.32 per 1,000 person-years). Taken together, these five cancers accounted for 67.6% of the VTE cases.

**Table 1:** **Site of cancer and associated incidence rate of VTE among all cancer patients**

		VTE (algorithm 1)^#^				VTE (algorithm 2)^&^			
	Total patient (n)	VTE cases (n)	Rate of VTE (%)	Observation time (p-y)^a^	Incidence of VTE (per 1,000 p-y)	VTE cases (n)	Rate of VTE (%)	Observation time (p-y)^a^	Incidence of VTE (per 1,000 p-y)
All patients	43,855	1,388	3.2	140,524	9.88	473	1.1	141,304	3.35
Male	23,115	896	3.9	66,063	13.56	259	1.1	66,571	3.89
Female	20,740	492	2.4	74,461	6.61	214	1.0	74,734	2.86
**Site of cancer**									
Liver	5,272	764	14.5	11,197	68.23	105	2.0	11,593	9.06
Pancreas	618	19	3.1	683	27.83	11	1.8	685	16.05
Lung	4,159	121	2.9	7,025	17.22	72	1.7	7,058	10.20
Multiple myeloma	183	4	2.2	375	10.65	3	1.7	379	7.92
Non-Hodgkin’s lymphoma	1,011	30	3.0	3,219	9.32	15	1.5	3,248	4.62
Leukemia	689	16	2.3	1,740	9.20	4	0.6	1,758	2.28
Renal	1,303	33	2.5	4,655	7.09	16	1.2	4,686	3.41
Sarcoma	458	12	2.6	1,761	6.82	9	2.0	1,773	5.08
Stomach	2,231	40	1.8	6,110	6.55	22	1.0	6,123	3.59
Ovary	668	15	2.3	2,390	6.28	11	1.7	2,405	4.57
Colorectum	6,462	112	1.7	22,452	4.99	69	1.1	22,502	3.07
Esophageal	761	6	0.8	1,239	4.84	4	0.5	1,240	3.23
Brain	522	7	1.3	1,559	4.49	5	1.0	1,562	3.20
Endometrium and cervix	2,327	43	1.9	10,024	4.29	31	1.3	10,055	3.08
Prostate	1,943	29	1.5	7,218	4.02	20	1.0	7,232	2.77
Bladder	1,723	23	1.3	6,477	3.55	14	0.8	6,484	2.16
Testis	119	2	1.7	582	3.44	0	0.00	590	0.00
Other abdominal	671	9	1.3	1,681	3.36	4	0.6	1,685	2.37
Skin	898	11	1.2	3,426	3.21	6	0.7	3,436	1.75
Hodgkin’s lymphoma	85	1	1.2	342	2.92	0	0.00	348	0.00
Head and neck	4,390	40	0.9	14,922	2.68	20	0.5	14,955	1.34
Breast	6,035	45	0.8	25,438	1.77	29	0.5	25,485	1.14
Thyroid	1,327	6	0.5	6,009	1.00	3	0.2	6,020	0.50

*Abbreviations*: p-y, person-years.

^a^For VTE cases, person-years were calculated from index date to the date of first hospitalization for VTE during or after cancer diagnosis. For patients without VTE event, person-years were calculated from index date until end of follow-up date.

^#^VTE algorithm 1 was defined as a hospital admission with diagnostic codes of VTE (ICD-9-CM codes: 415.1x, 451.xx, 452, and 453.xx).

^&^VTE algorithm 2 was based on both the hospital admission with diagnostic codes of VTE and managements of VTE (prescription of intravenous or subcutaneous (IV/SC) anticoagulants (unfractionated heparin (UFH) or low-molecular-weight heparin (LMWH)) or reimbursement codes of surgical thromboectomy) during the hospital stay.

Hospital admissions for VTE (algorithm 2) were identified in 473 patients (1.1%) during or after index date. The overall incidence rate of VTE (algorithm 2) was 3.35 per 1,000 person-years; slightly higher in men than women (3.89 vs. 2.86 per 1,000 person-years) (Table 1). The incidence rates of VTE were higher in certain cancers, particularly cancer of pancreas (16.05 per 1,000 person-years), lung (10.20 per 1,000 person-years), liver (9.06 per 1,000 person-years), multiple myeloma (7.92 per 1,000 person-years), and sarcoma (5.08 per 1,000 person-years). Taken together, these five cancers accounted for 42.3% of all VTE cases.

### Clinical characteristics of VTE events

Most VTE events (VTE algorithm 1) (53.5%) occurred within 90 days after index date, with 35.8% of VTE events occurring on the index date (Table 2). Median time-to-VTE was 70 days (range, 0–3,124 days). Among patients who experience a VTE, the cumulative occurrence of VTE within 30, 90, 180, 270, and 365 days after index date were 42.9%, 53.5%, 61.8%, 66.3%, and 70.8%, respectively. Among patients hospitalized for VTE (algorithm 2), 59.4% of VTE events occurred within 1 year after index date, with 18.0% of VTE events occurring on the index date (Table 2). Median time-to-VTE was 222 days (range, 0–3,124 days). Cumulative occurrence of VTE within 30, 90, 180, 270, and 365 days after index date were 25.2%, 39.8%, 47.8%, 53.9, and 59.4%, respectively.

**Table 2:** **Time-to-VTE after cancer diagnosis among all cancer patients**

	VTE algorithm 1^#^(N = 1,388)		VTE algorithm 2^&^(N = 473)	
Time-to-VTE	Patient no. (%)	Cumulative rate of VTE (%)	Patient no. (%)	Cumulative rate of VTE (%)
0 days	497 (35.8)	35.8	85 (18.0)	18.0
1 – 30 days	98 (7.1)	42.9	34 (7.2)	25.2
31 – 90 days	147 (10.6)	53.5	69 (14.6)	39.8
91 – 180 days	115 (8.3)	61.8	38 (8.0)	47.8
181 – 270 days	63 (4.5)	66.3	29 (6.1)	53.9
271 – 365 days	62 (4.5)	70.8	26 (5.5)	59.4
366 – 545 days	70 (5.0)	75.8	32 (6.8)	66.2
546 – 761 days	75 (5.4)	81.2	33 (7.0)	73.2
>731 days*	261 (18.8)	100.0	127 (26.9)	100.0

*The last observed events occurred 3,124 days after index date.

^#^VTE algorithm 1 was defined as a hospital admission with diagnostic codes of VTE (ICD-9-CM codes 415.1x, 451.xx, 452, and 453.xx).

^&^VTE algorithm 2 was based on both the hospital admission with diagnostic codes of VTE and managements of VTE (prescription of intravenous or subcutaneous (IV/SC) anticoagulants (unfractionated heparin (UFH) or low-molecular-weight heparin (LMWH)) or reimbursement codes of surgical thromboectomy) during the hospital stay.

Anatomic distribution of VTE is shown in Table 3. Among 1,388 patients with VTE (algorithm 1), 9.7% of patients had pulmonary embolism (PE) (with or without venous thrombosis) and 90.4% of patients had venous thrombosis. Among those with venous thrombosis, 52.9% had intra-abdominal thrombosis (thrombosis of renal, hepatic, or portal vein) and 27.7% had thrombosis of other unspecified site. Among 473 patients with VTE (algorithm 2), 16.1% had PE (with or without venous thrombosis) and 80.7% had venous thrombosis. Among those with venous thrombosis, 19.0% had intra-abdominal thrombosis and 53.9% had thrombosis of unspecified site.

**Table 3:** **Anatomic distribution of VTE among all cancer patients**

	VTE algorithm 1^#^	VTE algorithm 2^&^
	(N = 1,388)	(N = 473)
Sites	Patient no. (%)	Patient no. (%)
Pulmonary embolism	118 (8.5)	76 (16.1)
Pulmonary embolism and venous thrombosis	16 (1.1)	15 (3.2)
Thrombosis of extremities	73 (5.3)	19 (4.0)
Thrombosis of vena cava	38 (2.7)	12 (2.5)
Thrombosis of renal vein, hepatic vein or portal vein	734 (52.9)^a^	90 (19.0)^b^
Thrombosis of unspecified site	384 (27.7)	255 (53.9)
Superficial venous thrombosis	4 (0.3)	0 (0.0)
Multiple thrombotic sites	21 (1.5)^c^	6 (1.3)^d^

^a^715 patients had portal vein thrombosis, 12 patients had hepatic vein thrombosis, 7 patients had thrombosis of renal vein.

^b^86 patients had portal vein thrombosis, 2 patients had hepatic vein thrombosis, 2 patients had thrombosis of renal vein.

^c^16 patients had concomitant thrombosis of vena cava and intra-abdominal venous, 1 patient had concomitant thrombosis of extremities and other unspecified site, and 3 patients had concomitant thrombosis of vena cava, intra-abdominal venous and other unspecified site.

^d^4 patients had concomitant thrombosis of vena cava and intra-abdominal venous, and 2 patients had concomitant thrombosis of intra-abdominal venous and other unspecified site.

^#^VTE algorithm 1 was defined as a hospital admission with diagnostic codes of VTE (ICD-9-CM codes 415.1x, 451.xx, 452, and 453.xx).

^&^VTE algorithm 2 was based on both the hospital admission with diagnostic codes of VTE and managements of VTE (prescription of intravenous or subcutaneous (IV/SC) anticoagulants (unfractionated heparin (UFH) or low-molecular-weight heparin (LMWH)) or reimbursement codes of surgical thromboectomy) during the hospital stay.

### Baseline characteristics and risk factors for VTE

The mean age (± SD) of cancer patients with VTE (algorithm 2) was 60.9 years (±14.3 years), which was only slightly higher but statistically significantly different from cancer patients without VTE (59.5 ± 15.9 years) (Table 4). Compared with cancer patients without VTE, more cancer patients with VTE had prior histories of VTE within the 2 years before index date (1.1% vs. 0.2%, p < 0.0001). Furthermore, patients with VTE were significantly more likely to have comorbid diseases (including hypertension, heart failure, ischemic heart disease, renal insufficiency, liver disease, rheumatologic diseases, arterial embolism, obesity, and varicose veins of lower extremities) than patients without VTE. Compared with patients without VTE, more patients with VTE received major surgery, active therapy, and G-CSF, or were diagnosed with infectious diseases within 3 months before/during the VTE event. Hospital admission was more frequent in patients with VTE (60.0% vs. 32.1%, p < 0.0001). Among patients who received active therapy, more patients with VTE received chemotherapy (38.5% vs. 11.9%, p < 0.0001) and combination therapy (4.2% vs. 1.2%, p < 0.0001), but more patients without VTE received hormone therapy (7.3% vs. 3.6%, p < 0.0001).

**Table 4:** **Baseline characteristics of cancer patients with or without VTE**

	Patients without VTE		VTE (algorithm 2^&^)		P-value
	N = 43,382		N = 473		
Mean age (years)	59.52 ± 15.92		60.86 ± 14.26		0.0440*
	**No.**	**%**	**No.**	**%**	
Age groups (years)					0.0005*
≤18	502	1.2	1	0.2	
19 – 40	4,431	10.2	36	7.6	
41 – 60	16,847	38.8	179	37.9	
61 – 80	18,183	41.9	227	48.0	
≥81	3,419	7.9	30	6.3	
Gender					0.3695
Male	22,856	52.7	259	54.8	
Female	20,526	47.3	214	45.2	
Prior history of VTE	67	0.2	5	1.1	0.0011*
Hypertension	15,981	36.8	223	47.2	<0.0001*
Heart failure	1,793	4.1	31	6.6	0.0087*
Ischemic heart disease	6,005	13.8	86	18.2	0.0066*
Atrial fibrillation	649	1.5	9	1.9	0.4522
Renal insufficiency	3,270	7.5	53	11.2	0.0027*
Chronic lung disease	8,211	18.9	95	20.1	0.5229
Diabetes mellitus	7,874	18.2	97	20.5	0.1861
Stroke	2,690	6.2	30	6.3	0.8988
Degenerative & paralytic neurologic disease	3,665	8.5	47	9.9	0.2474
Rheumatologic diseases	573	1.3	12	2.5	0.0218*
Liver disease	7,959	18.4	119	25.2	0.0001*
Arterial embolism	165	0.4	7	1.5	0.0028*
Anemia	5,179	11.9	62	13.1	0.4354
Varicose veins of lower extremities	170	0.4	5	1.1	0.0418*
Peripheral vascular disease	515	1.2	9	1.9	0.1543
Pregnancy	74	0.2	2	0.4	0.1979
Infectious diseases	15,809	36.4	242	51.2	<0.0001*
Major trauma before VTE event	1260	2.9	18	3.8	0.2465
Major spine trauma	426	1.0	6	1.3	0.4775
Major extremity trauma	861	2.0	12	2.5	0.3924
Major surgery before VTE event	10,152	23.4	286	60.5	<0.0001*
CNS	457	1.1	8	1.7	0.1779
Thorax	4,706	10.9	158	33.4	<0.0001*
Abdomen	6,094	14.1	167	35.3	<0.0001*
Urogenital	1,397	3.2	43	9.1	<0.0001*
Orthopedic	164	0.4	1	0.2	1.0000
Hospitalization	13,909	32.1	284	60.0	<0.0001*
Blood transfusion	3,665	8.5	49	10.4	0.1376
Active therapy	9,079	20.9	221	46.7	<0.0001*
Chemotherapy only	5,151	11.9	182	38.5	<0.0001*
Radiation only	214	0.5	2	0.4	1.0000
Hormone therapy only	3,180	7.3	17	3.6	0.0020*
Combination therapy	534	1.2	20	4.2	<0.0001*
Use of erythropoietin stimulating agent (ESA)	828	1.9	9	1.9	0.9926
Use of granulocyte colony-stimulating factor (GCSF)	1,282	3.0	41	8.7	<0.0001*
Thalidomide therapy	27	0.1	0	0.0	1.0000
Obesity	146	0.3	5	1.1	0.0243*

*p-value < 0.05.

^&^VTE algorithm 2 was based on both the hospital admission with diagnostic codes of VTE and managements of VTE (prescription of intravenous or subcutaneous (IV/SC) anticoagulants (unfractionated heparin (UFH) or low-molecular-weight heparin (LMWH)) or reimbursement codes of surgical thromboectomy) during the hospital stay.

The results of multivariable logistic regression analysis showed that risk factors for VTE were primary cancer sites of GI, brain, lung, gynecologic, and renal, prior history of VTE, hypertension, arterial embolism, obesity, and rheumatologic diseases (Table 5). In addition, major thoracic, abdominal, and urogenital surgery, chemotherapy, and combination therapy were significantly associated with higher risk of VTE. In contrast, blood transfusion was associated with reduced risk of VTE.

**Table 5:** **Multivariable logistic regression: risk factors for VTE among all cancer patients**

Variable	Odds ratio	95% Cl
Cancer sites		
Low risk (reference)		
High risk	1.63	1.31 - 2.02
Hematologic	1.26	0.79 - 2.00
**Prior history of VTE**	4.32	1.60 - 11.66
**Comorbid diseases**		
Hypertension	1.41	1.17 - 1.70
Arterial embolism	2.96	1.31 - 6.67
Obesity	2.88	1.13 - 7.35
Rheumatologic diseases	1.90	1.05 - 3.43
**Potential risk factors**		
Surgery		
Thoracic	2.35	1.91 - 2.89
Abdominal	1.99	1.62 - 2.45
Urogenital	2.12	1.52 - 2.94
Chemotherapy	3.61	2.95 - 4.41
Combination therapy	4.95	3.08 - 7.96
Blood transfusion	0.57	0.42 - 0.77

### Treatment pattern of VTE

Among 1,388 patients hospitalized for VTE (algorithm 1), only 33.6% of patients received anticoagulant therapy or surgical thromboectomy during the hospital stay. Only 7.9% of patients with thrombosis of hepatic, portal, or renal vein alone (n = 734) received management of VTE. In contrast, excluding patients with superficial vein thrombosis (n = 4), anticoagulation or surgical thromboectomy was performed in 62.9% of patients with other sites of venous thrombosis or PE (n = 650). Among cancer patients with VTE events (algorithm 2), 1.5% of patients received thromboecytomy, and 98.5% of patients received LMWH/ UFH for initial treatment of VTE.

Patients with VTE (algorithm 2) were followed to analyze the long-term treatment of VTE. Among 473 patients with VTE (algorithm 2), 58 patients (12.3%) did not have any medical claim in the database after the VTE event. We therefore explored patterns of long-term anticoagulant treatment in the remaining 415 patients. Overall, long-term anticoagulant therapy was initiated in 64.1% of patients (Table 6). Among them, 46.3% of patients received warfarin alone and 9.6% of patients received LMWH at any time. The median duration of anticoagulant therapy was 66 days (range, 2–1,442 days). Among these patients, 58.7%, 18.4%, 13.5% and 9.4% of them received ≤ 3 months, 3–6 months, 6–12 months and ≥ 12 months of long-term anticoagulant therapy, respectively.

**Table 6:** **Initial and long-term treatment of VTE among all cancer patients**

	VTE (algorithm 1^#^)	VTE (algorithm 2^&^)
	(N = 1,388)	(N = 473)
**Initial treatment**	**Patient no. (%)**	**Patient no. (%)**
Thromboectomy	7 (0.5)	7 (1.5)
Low molecular weight heparin (LMWH)		
LMWH alone	58 (4.2)	64 (13.5)
LMWH + warfarin	143 (10.3)	148 (31.3)
Unfractioned heparin (UFH)		
UFH alone	79 (5.7)	115 (24.3)
UFH + warfarin	72 (5.2)	77 (16.3)
UFH + LWMH		
UFH + LMWH	16 (1.1)	17 (3.6)
UFH + LMWH + warfarin	43 (3.1)	45 (9.5)
Warfarin only	49 (3.5)	0 (0.00)
No anticoagulation therapy	921 (66.4)	0 (0.00)
**Long-term treatment**	**Patient no. (%)**	**Patient no. (%)**
LMWH alone	-	24 (5.8%)
UFH alone	-	24 (5.8%)
Warfarin alone	-	192 (46.3%)
LMWH + warfarin	-	13 (3.1%)
UFH + warfarin	-	10 (2.4%)
LMWH and UFH	-	3 (0.7%)
No anticoagulant therapy	-	149 (35.9%)

^#^VTE algorithm 1 was defined as a hospital admission with diagnostic codes of VTE (ICD-9-CM codes 415.1x, 451.xx, 452, and 453.xx).

^&^VTE algorithm 2 was based on both the hospital admission with diagnostic codes of VTE and managements of VTE (prescription of intravenous or subcutaneous (IV/SC) anticoagulants (unfractionated heparin (UFH) or low-molecular-weight heparin (LMWH)) or reimbursement codes of surgical thromboectomy) during the hospital stay.

## Discussion

Using the NHI research database, we examined the incidence, risk factors and clinical characteristics of VTE among patients with different cancer types over a period of 9 years. The major strength of our study is that we report population-based rates for VTE incidence and treatment patterns in an Asian population. Two alternative algorithm were used to capture VTE diagnosis codes and managements of VTE during the hospital stay. The second algorithm was similar to the outcome definition used in previous population-based studies [18-20]. To avoid serious underestimation of the incidence rate of VTE among cancer patients, we also included patients who hospitalized for VTE only (algorithm 1). By adopting two VTE algorithms in our study, we believe we could provide crude incidence rates of accidentally detected VTE and clinical symptomatic VTE.

In our study, 1.1% to 3.2% of all newly diagnosed cancer patients were hospitalized for VTE events, based on two algorithms of VTE. Our study showed a higher incidence of VTE among the non-Caucasian cancer patients, which is consistent with Yu et al. [20]. The incidence rate of VTE is 21- to 62- folds among cancer patients than the general population in Taiwan (15.9 per 100,000 person-years) [19]. Nevertheless, the VTE incidence is significantly lower than reports among Caucasian populations [21-23]. Among Caucasian patients with cancer, the estimated incidence rate of VTE ranges from 0.6% to 12.1% [24-31]. The incidence rate in our study is lower than reported rates of Cronin-Fenton et al. [29] and Khorana et al. [28] among Caucasian populations.

There is convincing evidence that the risk of VTE increases in proportion to the number of predisposing factors [32,33]. Identification of risk factors can help us to identify cancer patients with higher risk for VTE and optimize the prophylaxis of VTE. In our study, an increased risk of VTE was associated with cancer site, prior history of VTE, arterial embolism, hypertension, obesity, major surgery, chemotherapy, and combination therapy. These results were consistent with existing studies among cancer patients [25,27,28,31,34-36]. Rheumatologic diseases were first identified as an independent risk factor for VTE among cancer patients in our study. Recent epidemiological studies among general population also suggested that certain rheumatologic diseases, including rheumatic arthritis (RA), dermatomyositis/polymyositis and systemic lupus erythematous (SLE) were associated with increased risk of VTE [37,38].

Thrombosis of portal vein is common in our study. In our study, 51.5% (algorithm 1) and 18.2% (algorithm 2) of patients with VTE had portal vein thrombosis alone. More than 80% of the portal vein thrombosis occurred in patients with liver cancer. Consistent with previous studies in Korea [12,13], we found that most patients with thrombosis of renal, portal or hepatic veins did not received anticoagulant treatment in clinical practice. Liver cancer is a well-known risk factor for portal vein thrombosis [39,40]. The high prevalence of liver cancer in Taiwan [41] may result in the high prevalence of portal vein thrombosis in our study cohort. However, there is no randomized trial to guide the use of anticoagulation in this situation [39,42]. Given the common prevalence of portal vein thrombosis, further studies are needed to clarify the role and duration of anticoagulant therapy in these patients.

This study represents the largest national population-based epidemiologic study in Asia that described the management of VTE among cancer patients in Taiwan. Our study found that the adherence to treatment guidelines was poor in Taiwan. Long-term anticoagulant therapy was only initiated in 64.1% of patients with VTE (algorithm 2). In existing clinical guidelines, 3–6 months of LMWH are recommended for long-term treatment of VTE in cancer patients [1,6,7,42]. However, in our study, among patients who received long-term treatment of VTE, LMWH was administered to only 9.6% of patients at anytime following the VTE events. Most patients received warfarin monotherapy for the long-term treatment of VTE. This is probably because reimbursement for outpatient use of LMWH by NHI was limited to pregnant patients with prosthetic valve replacement [20]. Furthermore, in our study, treatment duration of long-term anticoagulant therapy was shorter than those recommended in clinical guidelines [1,6,7,42].

Some limitations exist in the present study, generally related to the use of a claims database. First, the actual incidence of VTE may be underestimated, because some patients with PE die suddenly without accurate diagnosis. Patients who treated in the outpatient clinic using LMWH/oral anticoagulants were not included based on our definitions. Identification of a VTE event based on admission record may underestimate the incidence of VTE. However, the magnitude of underestimation could be very small as home injection of LMWH/UFH/fondapariunx is not reimbursed under Taiwan’s National Health Insurance system, cancer patients usually are admitted for receiving these treatments. In addition, as patients usually receive LMWH/UFH for the initial treatment of VTE (“incident” VTE in our study) and warfarin for the long-term treatment, it is easier for physicians to monitor activated partial thromboplastin time (aPTT) and international normalized ratio (INR) when patients are hospitalized. These could be supported by another epidemiological study of VTE in the Taiwanese general population, in which the authors define their VTE cases as those who admitted for a VTE event [19]. Furthermore, patients with asymptomatic VTE may not be documented. We were unable to distinguish symptomatic VTE and incidentally detected VTE as the information were not routinely captured in a claim database. However, our estimates provide the incidence rate of clinically overt VTE, which is useful in helping decision making of VTE prophylaxis in clinical practice. A second limitation of our study is we identified our cancer cases based on a diagnosis of cancer at hospitalization, cancer cases diagnosed based on outpatient visit were not included, which may underestimate the cancer population. Nevertheless, as all NHI beneficiaries diagnosed with cancer is required to have an confirmed cancer diagnosis from the hospital to be eligible for a Certificate of Catastrophic Illness to be exempted from all co-payments, cancer cases who only be treated in outpatient setting are very few in Taiwan.

This approach could thus help us precisely identify the cancer cases and avoid potential misclassifications. Third, other factors that may contribute to development of VTE, including disease stage, laboratory data and performance status could not be obtained from the databases. However, we include many variables such as active therapy (e.g. radiation therapy) to serve as the proxy of disease severity to reduce potential confounding effects. Fourth, the study cohort may have received anticoagulant treatment based on certain baseline and prognostic characteristics. Finally, thromboprophylaxis can alter the incidence of VTE, but its use in our study population is unknown.

## Conclusion

In conclusion, this retrospective cohort study describes the epidemiology, risk factors, and clinical profile of VTE across different types of cancer among an Asian population. The incidence rate of VTE was higher among cancer patients than among non-cancer patients in Taiwan but lower than among Caucasian cancer patients. Clinical practitioners should carefully monitor patients with cancer for VTE. Adherence to treatment guidelines was low in this real world cohort of Taiwanese cancer patients with VTE. Treatment and prophylaxis of VTE should be optimized, especially in patients with higher-risk of VTE. Due to the different epidemiologic profile of VTE in Taiwan compared with Caucasian population, further investigations are desired to estimate the harms and benefits of anticoagulants treatment and thromboprophylaxis among Asian cancer patients.
